# Diarrhoea, enteric pathogen detection and nutritional indicators among controls in the Global Enteric Multicenter Study, Kenya site: an opportunity to understand reference populations in case–control studies of diarrhoea

**DOI:** 10.1017/S0950268818002972

**Published:** 2018-11-15

**Authors:** D. M. Berendes, C. E. O'Reilly, S. Kim, R. Omore, J. B. Ochieng, T. Ayers, K. Fagerli, T. H. Farag, D. Nasrin, S. Panchalingam, J. P. Nataro, K. L. Kotloff, M. M. Levine, J. Oundo, K. Laserson, R. F. Breiman, E. D. Mintz

**Affiliations:** 1 School of Civil and Environmental Engineering, Georgia Institute of Technology, Atlanta, Georgia, USA; 2 Division of Foodborne, Waterborne, and Environmental Diseases, Centers for Disease Control and Prevention (CDC), Atlanta, GA, USA; 3 Kenya Medical Research Institute, Center for Global Health Research (KEMRI-CGHR), Kisumu, Kenya; 4 Center for Vaccine Development, University of Maryland School of Medicine, Baltimore, MD, USA; 5 Institute for Health Metrics and Evaluation, University of Washington, Seattle, WA, USA; 6 Department of Pediatrics, University of Virginia School of Medicine, Charlottesville, VA, USA; 7 KEMRI/CDC, Kisumu, Kenya; 8 CDC India, Delhi, India; 9 CDC-Kenya, Nairobi, Kenya; 10 Emory Global Health Institute, Emory University, Atlanta, Georgia, USA

**Keywords:** Diarrhoea, estimating disease prevalence, gastrointestinal infections

## Abstract

Given the challenges in accurately identifying unexposed controls in case–control studies of diarrhoea, we examined diarrhoea incidence, subclinical enteric infections and growth stunting within a reference population in the Global Enteric Multicenter Study, Kenya site. Within ‘control’ children (0–59 months old without diarrhoea in the 7 days before enrolment, *n* = 2384), we examined surveys at enrolment and 60-day follow-up, stool at enrolment and a 14-day post-enrolment memory aid for diarrhoea incidence. At enrolment, 19% of controls had ⩾1 enteric pathogen associated with moderate-to-severe diarrhoea (‘MSD pathogens’) in stool; following enrolment, many reported diarrhoea (27% in 7 days, 39% in 14 days). Controls with and without reported diarrhoea had similar carriage of MSD pathogens at enrolment; however, controls reporting diarrhoea were more likely to report visiting a health facility for diarrhoea (27% *vs.* 7%) or fever (23% *vs.* 16%) at follow-up than controls without diarrhoea. Odds of stunting differed by both MSD and ‘any’ (including non-MSD pathogens) enteric pathogen carriage, but not diarrhoea, suggesting control classification may warrant modification when assessing long-term outcomes. High diarrhoea incidence following enrolment and prevalent carriage of enteric pathogens have implications for sequelae associated with subclinical enteric infections and for design and interpretation of case–control studies examining diarrhoea.

## Introduction

Globally, over 1.7 billion children are affected each year by diarrhoea [1], an important – yet complex – health condition. Across numerous published studies [2], measurement of diarrhoea varies from self-report to confirmed clinical and laboratory diagnoses [3]. Even the most detailed studies fail to identify the aetiologic agent in all cases, but clinical and laboratory data now exist to estimate pathogen-specific disease burdens. Diarrhoea can be caused by various infectious agents – bacteria, viruses, protozoa and soil-transmitted helminths – that differ in their relative contribution to diarrhoeal morbidity and mortality [3–5]. These organisms also vary in their incubation period, the probability with which symptoms occur following exposure, and the duration during which the organism is excreted in faeces after symptoms resolve [6]. During epidemiologic studies of diarrhoeal diseases, these variations make it difficult to accurately identify unexposed controls and to identify the precise cause of acute symptoms when multiple pathogens are identified in stool testing.

Case–control studies with laboratory testing of stool specimens are common designs for ascertaining aetiologic agents [7–10] and assessing pathogen-specific disease burden and risk factors [11, 12]. Case and control definitions that employ specific clinical criteria allow for more accurate classification of disease severity and health status, and a more precise outcome measure [13]. Often control eligibility is restricted by clinical criteria, such as the absence of diarrhoeal symptoms in the control for a defined period. As mild diarrhoeal illness in young children is common in developing countries, imperfect recall may lead to misclassification of children convalescing from an episode of diarrhoeal disease or incubating diarrhoeal disease as controls [14–16]. Moreover, as cases are often enrolled in health facilities while controls are enrolled in the community, specimen collection from controls and transport to a laboratory for confirmation of control (non-diseased) status is challenging and may yield a higher proportion of false-negative tests, given that asymptomatic individuals often produce fewer pathogens per gram of stool [6]. Further, logistical constraints in case–control studies often restrict contact with controls to a single visit at enrolment, where both inclusion criteria and risk factors are ascertained [9–11]. Follow-up to confirm disease-free status is rarely attempted.

Because of the challenges in accurately identifying unexposed controls in case–control studies of diarrhoea, and the growing recognition of subclinical enteric infections as a determinant of longer term health outcomes, we sought to examine the incidence of diarrhoea, subclinical enteric infections and growth stunting within a reference population. The Global Enteric Multicenter Study (GEMS) – a multisite case–control study of moderate-to-severe diarrhoea (MSD) in children <5 years old in Africa and south Asia [17] – provides a unique opportunity to examine diarrhoea incidence, enteric pathogen prevalence and longer term outcomes including growth stunting, in a control population. The goal of this study was to characterise the health of controls in the GEMS study following enrolment, including diarrhoeal symptoms, enteric pathogen detection in stool and stunting. Studying controls can reveal background rates of diarrhoea and enteric pathogen carriage, and inform future criteria for control selection in diarrhoeal disease studies.

## Methods

GEMS was a matched case–control study of MSD in children <5 years old, conducted in seven sites in sub-Saharan Africa and South Asia to improve understanding of the aetiology and burden of diarrhoeal diseases in low-income settings [17]. This analysis focuses on GEMS data collected at the Kenya study site [5, 18–21].

### Study site

The GEMS Kenya site, located in rural, western Kenya, has been described previously [13, 18–21]. The population enrolled at the Kenya site participated in a health and demographic surveillance system (HDSS) that visited each household thrice annually to obtain information about births, deaths, migration and other factors. Children were enrolled between 31 January 2008 and 29 January 2011 and between 31 October 2011 and 30 September 2012.

### Inclusion criteria for controls

Control children matched by age, sex and neighbourhood were randomly selected from the HDSS population and visited at home within 14 days of case identification. Controls were enrolled if their caretaker reported the child was free of diarrhoea for 7 days before the visit, and consented to participation. Detail on sampling frame and case–control selection are described elsewhere [13].

### Enrolment and follow-up

At enrolment, a questionnaire was administered to determine each child's eligibility as a control. A stool specimen was obtained from each eligible consented child, delivered to the laboratory and processed within 18 h of enrolment. A questionnaire concerning household demographics; socio-economic status; water, sanitation and hygiene (WASH) conditions; and feeding and other medical conditions of the child was administered to caretakers, and the child's length/height was measured. Finally, the caretaker was given a 14-day memory aid form to record daily diarrhoeal incidence and was instructed that enumerators would return in approximately 60 days (acceptable window: 49–91 days) to conduct a follow-up visit. At the 60-day visit, the memory aid form was collected, data on illness and healthcare seeking for the child subsequent to enrolment were collected, and anthropometric measurements were repeated.

### Stool collection at enrolment

All stool specimens from controls underwent the same methods of collection, transport, delivery to the laboratory and testing for the spectrum of bacterial, viral and parasitic enteric pathogens via conventional microbiological methods as specimens from cases [22].

### Administration of the 14-day memory aid form

A memory aid for daily incidence of diarrhoea was created for the caretaker of cases and control children to complete during the 14 days following enrolment [13, 18]. Caretakers were trained in the definition of diarrhoea used – passage of ⩾3 loose or watery stool in the previous 24 h – and instructed to fill the form daily. At the 60-day visit, the memory aid was reviewed with the caretaker to resolve any unclear or missing data. We defined ‘any diarrhoea’ as ⩾1 day of diarrhoea denoted on the memory aid within the 14-day period after enrolment. Incidence was also broken down by date of onset post-enrolment.

### Anthropometry

Anthropometric measurements (length/height) were collected for controls at home at enrolment and follow-up as described previously [13] using a ‘Shorr board’. Height-for-age *Z*-scores (HAZ) were calculated using a WHO SAS macro and the WHO Child Growth Standards for the reference population [23, 24]. Staff performing measurements underwent a training and quality assessment regimen for the duration of the study, as previously described [13]. To mitigate the impact of measurement error, outliers defined by both WHO [24] and using median absolute deviation methods [25] were censored. HAZ scores were calculated to assess stunting for each child at enrolment and 60-day follow-up based on standard WHO stunting criteria (<−2 *z*-scores).

### Statistical analysis

Data were stored and managed in SAS software version 9.4 (SAS, Cary, NC, USA) and analyses conducted in R version 3.2.1 (R Foundation for Statistical Computing, Vienna, Austria [26]). We performed logistic regression to compare diarrhoea and enteric pathogen detection in controls. We categorised controls by (a) development of any/no reported diarrhoea (from memory aid data); (b) detection in stool collected at enrolment of any/no MSD enteric pathogen (defined as pathogens significantly associated with MSD at the Kenya site – rotavirus, *Cryptosporidium*, *Shigella* spp., typical enteropathogenic *Escherichia coli* (tEPEC), heat-stable-toxin-producing enterotoxigenic *E. coli* (ST-ETEC) and non-typhoidal *Salmonella* spp. [5]); (c) detection in stool at enrolment of any/no potential enteric pathogens (defined as any pathogens tested from the entire list of GEMS pathogens assessed in the stool specimen at enrolment, listed in Table S2 [22]); and (d) four distinct groups based on diarrhoea and MSD pathogen detection: diarrhoea + /pathogen + (G1), diarrhoea − /pathogen + (G2), diarrhoea + /pathogen − (G3) and diarrhoea − /pathogen − (G4) groups (Table S1b). G1 and G3 were combined in subsequent analyses to measure children with diarrhoea against children without reported diarrhoea but with pathogens detected (G2) and children without reported diarrhoea or pathogens detected (G4).

Logistic regression models were run with dummy variables for levels of the previously described subgroups as predictors, and clinical, health, WASH conditions and stunting as outcomes to investigate differences between groups. Age group (0–11, 12–23, 24–59 months) and sex were considered potential effect modifiers in all models and reported if significant at *α* = 0.05. Age groups and sex were included in models when effect modification was not present (all *P*-values for interaction with age or sex >0.05) and adjusted estimates are reported.

At the GEMS Kenya site, 125 (4.9%) controls were enrolled more than once. To examine their influence on the results, sensitivity analyses excluding repeat enrolments were conducted.

### Ethics

The study was reviewed and approved by the KEMRI Scientific and Ethical Review Committees (Protocol #1155) and the Institutional Review Board (IRB) at the University of Maryland, School of Medicine, Baltimore, MD, USA (UMD Protocol #H-28327). The IRB for CDC, Atlanta, GA, USA deferred its review to the University of Maryland IRB (CDC Protocol #5038).

## Results

### Demographics, diarrhoea and detection of enteric pathogens in controls

**Fig. 1.**
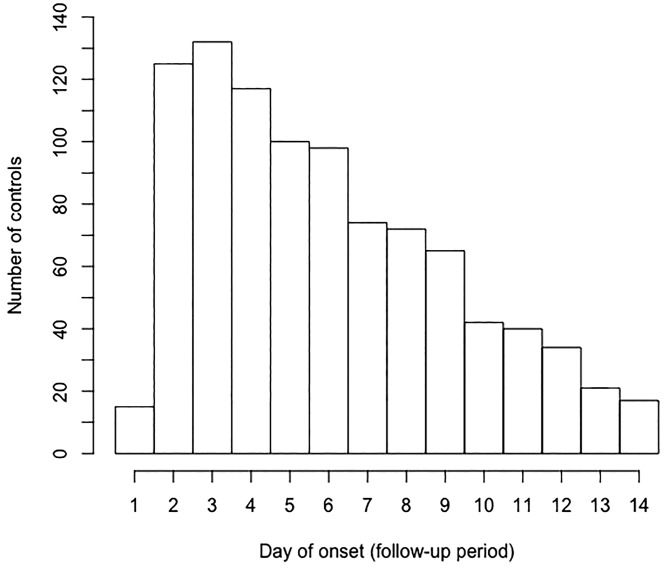
Date of onset of diarrhoea during 14-day memory aid period among controls with reported diarrhoea.

Of the 2534 controls in the GEMS Kenya site with follow-up during the acceptable window, 2384 (94%) had a completed memory aid for diarrhoea recall; we excluded from further analysis the 150 (6%) who did not (Table S1a). Among controls with a completed memory aid, mean age was 18 months [range 0–59 months old (mo)], 36% were infants (0–11 mo) and 57% were male (Table 1). Controls that did not have a completed memory aid form did not differ significantly in age or sex from those included (data not shown). Among the 919 (39%) controls that developed ‘any diarrhoea’, onset clustered soon after enrolment and peaked on day 3 [132 (14%), Fig. 1], with 643 (27% of all controls, 70% of controls with diarrhoea) reporting onset by day 7 (Table 1). Children 24–59 mo had lower reported diarrhoea incidence (31%) than those 0–11 or 12–23 mo (42%).

**Table 1.** Demographics, diarrhoea and enteric pathogen detection in control children^a^ at enrolment, Global Enteric Multicenter Study, Kenya site

Attribute	0–11 months old	12–23 months old	24–59 months old	Total (%)
Age group	856 (35.9%)	794 (33.3%)	734 (30.8%)	2384 (100%)
Male	506 (59.1%)	456 (57.4%)	386 (52.6%)	1348 (56.5%)
Any diarrhoea within 7 days after enrolment	260 (30.3%)	211 (26.6%)	172 (23.4%)	643 (27.0%)
Any diarrhoea within 14 days after enrolment	359 (41.9%)	330 (41.6%)	230 (31.3%)	919 (38.6%)
Days with diarrhoea among all controls, median (range)	0 (0–11)	0 (0–13)	0 (0–11)	0 (0–13)
Among only controls with diarrhoea, median (range)	3 (1–11)	3 (1–13)	3 (1–11)	3 (1–13)
MSD enteric pathogen^b^ detected in stool at enrolment	192 (22.4%)	171 (21.5%)	97 (13.2%)	460 (19.3%)
#MSD enteric pathogens^b^ detected in stool at enrolment, median (range)	0 (0–3)	0 (0–2)	0 (0–2)	0 (0–3)
Any potential enteric pathogen^c^ detected in stool at enrolment	597 (69.7%)	563 (70.9%)	469 (63.9%)	1629 (68.3%)
#Potential enteric pathogens^c^ detected in stool at enrolment, median (range)	1 (0–5)	1 (0–5)	1 (0–5)	1 (0–5)

a *N* = 2534 children enrolled as controls; however, *n* = 2384 control children that submitted a completed 14-day memory aid form.

b Any pathogens detected in a child's stool specimen at enrolment that were significantly associated with moderate-to-severe diarrhoea (MSD) at the GEMS Kenya site (Rotavirus, *Cryptosporidium*, *Shigella* spp., typical enteropathogenic *E. coli* (tEPEC), heat-stable-toxin-producing enterotoxigenic *E. coli* (ST-ETEC) and non-Typhoidal *Salmonella* spp.) [5].

c Any pathogens detected from the entire list of potential pathogens assessed in GEMS (Table S2) [22].

At least one MSD enteric pathogen was detected in 460 stool specimens collected from controls at enrolment (19%); detection rates decreased by age group (Table 1). The most prevalent MSD pathogens were tEPEC (4.8%), ST-ETEC (4.2%) and *Cryptosporidium* (4.1%, Table S2). Co-detection of MSD pathogens was uncommon (2%). Approximately 68% of controls’ stool specimens at enrolment had at least one potential enteric pathogen detected, most commonly *Giardia* spp. (24%) and enteroaggregative *E. coli* (16%, Table S2).

Adjusting for age and sex, detection of tEPEC was higher in controls that developed diarrhoea than in those that did not (OR 1.5, 95% CI 1.0–2.1, *P* = 0.05, Table S2).

### Health outcomes and WASH exposures by diarrhoea and pathogen detection

Controls that did and did not develop any diarrhoea did not vary significantly in detection of any MSD enteric pathogens in stool collected at enrolment (Table 2). Controls that developed diarrhoea had significantly higher odds of reporting fever in the week preceding enrolment (OR 1.6, 95% CI 1.4–1.9) and of having used an unimproved water source (OR 1.3, 95% CI 1.1–1.5) than controls that did not develop diarrhoea. At 60-day follow-up, controls that developed diarrhoea had significantly higher odds of having visited a health facility for diarrhoea (OR 4.9, 95% CI 3.8–6.4), having had fever (OR 1.8, 95% CI 1.5–2.2) and having visited a health facility for fever (OR 1.5, 95% CI 1.2–1.9). Overall, 71% (253) of controls who reported having sought care for diarrhoea at the 60-day follow-up visit had reported diarrhoea on the memory aid. Only 101 (7%) of the 1465 controls who did not report diarrhoea on the memory aid reported having sought care for diarrhoea at the 60-day follow-up visit. Male controls that developed diarrhoea were significantly more likely to report having had dysentery in the last 60 days (OR 16.9, 95% CI 2.2–132), but female controls were not.

**Table 2.** Analysis of controls with/without any diarrhoea reported in 14-day memory aid form, Global Enteric Multicenter Study, Kenya site

Parameter	Controls with any diarrhoea^a^ *N* = 919	Controls without any diarrhoea^a^ *N* = 1465	aOR^b^	*P* value^b^
(a) Health conditions at enrolment				
Detection of an MSD enteric pathogen^c^ in stool	198 (21.5%)	262 (17.9%)	1.20 (0.97, 1.47)	0.090
Median #MSD enteric pathogens^c^ detected (interquartile range)^d^	0 (0–0)	0 (0–0)	1.16 (0.97, 1.39)	0.098
Detection of any potential enteric pathogen^e^ in stool	635 (69.1%)	993 (67.8%)	1.03 (0.86, 1.23)	0.784
Median #potential enteric pathogens^e^ detected (interquartile range)^d^	1 (0–2)	1 (0–2)	1.02 (0.94, 1.10)	0.709
Blood in stool collected	0	4 (0.3%)	–	–
Blood in stool (in last 7 days)	2 (0.2%)	4 (0.3%)	0.79 (0.11, 4.09)	0.783
Fever (in last 7 days)	401 (43.6%)	480 (32.8%)	**1.62 (1.37, 1.93)**	**< 0.001**
Vomiting (in last 7 days)	33 (3.6%)	36 (2.5%)	1.48 (0.91, 2.40)	0.115
(b) Water, sanitation and hygiene conditions at enrolment				
Any sanitation facility present	697 (75.8%)	1106 (75.5%)	1.04 (0.86, 1.26)	0.708
Unimproved water source^f^	361 (39.3%)	492 (33.6%)	**1.25 (1.05, 1.49)**	**0.010**
Water treated	521 (56.7%)	820 (56.0%)	1.02 (0.86, 1.20)	0.828
Water treated effectively^g^	496 (54.0%)	771 (52.6%)	1.05 (0.89, 1.23)	0.603
Water treated with chlorine	433 (47.1%)	648 (44.2%)	1.13 (0.95, 1.33)	0.158
(c) Health at 60-day follow-up				
Visited health facility for diarrhoea in last 60 days	253 (27.5%)	101 (6.9%)	**4.92 (3.84, 6.36)**	**<0.001**
Dysentery in last 60 days	16 (1.7%)	8 (0.6%)		
Females			1.34 (0.42, 4.25)	0.625
Males			**16.9 (2.18, 132)**	**0.007**
Visited health facility for dysentery in last 60 days	8 (0.9%)	4 (0.3%)	3.27 (1.02, 12.3)	0.055
Fever in last 60 days	606 (66.2%)	745 (51.3%)	**1.83 (1.54, 2.17)**	**< 0.001**
Visited health facility for fever in last 60 days	209 (22.7%)	234 (16.0%)	**1.52 (1.23, 1.87)**	**< 0.001**
Death of child	8 (0.9%)	5 (0.3%)	2.30 (0.76, 7.66)	0.146

**Bold** indicates significant at 0.05.

a Based on responses in 14-day memory aid.

b Adjusted for age group and sex, stratified estimates by age group or sex presented where effect modification significant at 0.05 was observed.

c Any pathogens detected in a child's stool specimen at enrolment that were significantly associated with moderate-to-severe diarrhoea (MSD) at the GEMS Kenya site [5].

d Modelled by multivariable Poisson regression. All other estimates by multivariable logistic regression.

e Any pathogens detected from the entire list of potential pathogens assessed in GEMS [22].

f Water source that does not meet the criteria for ‘improved’, per the Joint Monitoring Program criteria [40] of a source that is safely protected from outside contamination (especially faeces) via its construction or intervention.

g Effective water treatment classified as solar disinfection, chlorine disinfection, boiling or filtration through ceramic or other filter. Ineffective water treatment classified as filtration through a cloth, alum or other chemical added.

Although few deaths (13) were observed in control children, those with MSD pathogens detected in stool at enrolment were more likely to have died by 60-day follow-up than those without MSD pathogens [6/460 (1.3%) *vs.* 7/1924 (0.4%), OR 3.2, 95% CI 1.0–9.7, Table 3]. Five of six deaths in control children with MSD pathogens were among those who reported developing diarrhoea (data not shown). No other significant differences were observed between controls with/without MSD pathogens detected. Controls with and without any potential enteric pathogens detected in stool at enrolment did not differ significantly in health or WASH conditions at enrolment, or health at 60-day follow-up (Table S4).

**Table 3.** Analysis of controls with/without MSD enteric pathogen detected in stool at enrolment, Global Enteric Multicenter Study, Kenya site

Parameter	⩾1 MSD enteric pathogens^a^ detected *N* = 460 (%)	0 MSD enteric pathogens^a^ detected *N* = 1924 (%)	aOR^b^	*P* value^b^
(a) Health conditions at enrolment				
Blood in stool collected	0 (0)	4 (0.2)		
Blood in stool (in last 7 days)	1 (0.2)	5 (0.3)		
Fever (in last 7days)	179 (38.9)	702 (36.5)	1.12 (0.91, 1.38)	0.287
Vomiting (in last 7days)	16 (3.5)	53 (2.8)	1.23 (0.67, 2.14)	0.474
(b) Water, sanitation and hygiene conditions at enrolment				
Any sanitation facility present	359 (78.0)	1444 (75.1)	1.20 (0.94, 1.54)	0.139
Unimproved water source^c^	167 (36.3)	686 (35.7)	1.00 (0.81, 1.24)	0.998
Water treated	276 (60.0)	1065 (55.4)	1.20 (0.97, 1.48)	0.091
Water treated effectively^d^	258 (56.1)	1009 (52.4)	1.15 (0.93, 1.41)	0.197
Water treated with chlorine	221 (48.0)	860 (44.7)	1.15 (0.94, 1.41)	0.184
(c) Health at 60 days follow-up				
Diarrhoea	201 (44.2)	772 (40.4)	1.08 (0.88, 1.33)	0.465
Visited health facility for diarrhoea in last 60 days	77 (16.7)	277 (14.4)	1.08 (0.81, 1.41)	0.608
Dysentery in last 60 days	5 (1.1)	19 (1.0)	1.16 (0.38, 2.92)	0.772
Visited health facility for dysentery in last 60 days	1 (0.2)	11 (0.6)	0.37 (0.02, 1.92)	0.341
Fever in last 60 days	254 (55.8)	1097 (57.3)	0.92 (0.75, 1.13)	0.416
Visited health facility for fever in last 60days	79 (17.2)	364 (18.9)	0.85 (0.65, 1.11)	0.243
Death of child	6 (1.3)	7 (0.4)	**3.20 (1.02, 9.72)**	**0.038**

**Bold** indicates ORs significant at 0.05.

a Any pathogens detected in a child's stool specimen at enrolment that were significantly associated with moderate-to-severe diarrhoea (MSD) at the GEMS Kenya site [5].

b All adjusted ORs (aORs) adjusted for age group and sex of control child.

c Water source that does not meet the criteria for ‘improved’, per the Joint Monitoring Program criteria [40] of a source that is safely protected from outside contamination (especially faeces) via its construction or intervention.

d Effective water treatment classified as solar disinfection, chlorine disinfection, boiling or filtration through ceramic or other filter. Ineffective water treatment classified as filtration through a cloth, alum or other chemical added.

### Differences in health conditions in controls by diarrhoea-enteric pathogen group

When controls were divided by both reported diarrhoea and MSD pathogen detection, 198 (8.3%) reported diarrhoea and had an MSD pathogen detected (G1), 262 (11%) did not report diarrhoea but had an MSD pathogen detected (G2), 721 (30%) reported diarrhoea but did not have an MSD pathogen detected (G3), and 1203 (51%) did not report diarrhoea or have an MSD pathogen detected (G4, Table S1b). G1 and G3 controls tended to have similar health conditions when measured descriptively (Table S5). Differences in clinical conditions were assessed for combined diarrhoeal controls (G1 + G3 controls), non-diarrhoeal controls with MSD pathogens detected (G2) and non-diarrhoeal controls without MSD pathogens detected (G4, Table 4). G1 + G3 controls had significantly higher odds of having a fever (OR 1.7, 95% CI 1.4–2.0) or vomiting (OR 1.7, 95% CI 1.0–3.0) in the 7 days preceding enrolment compared with G4 controls.

**Table 4.** Differences in health and WASH conditions among controls by MSD pathogen detection in stool and reported diarrhoea, Global Enteric Multicenter Study, Kenya site^a^

Parameter	G1 + G3^b^ Diarrhoea aOR (95% CI)^c^ *n* = 919	G2 No diarrhoea, ⩾1 MSD pathogens^c^ detected aOR (95% CI)^d^ *n* = 262	G4 No diarrhoea, 0 MSD pathogens^c^ detected aOR (95% CI)^d^ *n* = 1203
Health at enrolment			
Fever (in last 7 days)	**1.68 (1.40–2.01)**	1.20 (0.90–1.58)	Ref.
Vomiting (in last 7 days)	**1.74 (1.03–2.99)**	1.96 (0.91–3.96)	Ref.
WASH conditions at enrolment			
Any sanitation facility present	1.09 (0.90–1.34)	1.36 (0.99–1.91)	Ref.
Unimproved water source^e^			Ref.
0–11 mo	1.12 (0.83–1.50)	0.96 (0.61–1.50)	
12–23 mo	1.16 (0.85–1.58)	**1.95 (1.22–3.11)**	
24–59 mo	0.93 (0.67–1.30)	0.58 (0.33–1.03)	
Water treated	1.04 (0.87–1.23)	1.22 (0.93–1.61)	Ref.
Water treated effectively^f^			Ref.
0–11 mo	1.07 (0.76–1.52)	0.85 (0.50–1.42)	
12–23 mo	1.00 (0.70–1.42)	**1.94 (1.09–3.45)**	
24–59 mo	1.21 (0.83–1.78)	1.15 (0.62–2.14)	
Water treated with chlorine			Ref.
0–11 mo	1.27 (0.90–1.79)	1.02 (0.60–1.72)	
12–23 mo	1.07 (0.75–1.53)	**1.93 (1.11–3.37)**	
24–59 mo	1.24 (0.85–1.82)	1.23 (0.66–2.28)	
Health at 60-day follow-up			
Visited health facility for diarrhoea	**4.77 (3.67–6.27)**	0.84 (0.47–1.41)	Ref.
Dysentery	**3.77 (1.53–10.6)**	1.62 (0.24–7.09)	Ref.
Visited health facility for dysentery	**—**	—	
Fever	**1.77 (1.48–2.12)**	0.85 (0.65–1.12)	Ref.
Visited health facility for fever	**1.46 (1.17–1.82)**	0.81 (0.54–1.17)	Ref.

**Bold** indicates significant at 0.05.

a Multivariable logistic regression models used for all.

b G1 and G3 were combined to represent all control children for whom diarrhoea was reported in the 14 days following enrolment.

c Any pathogens detected in a child's stool specimen at enrolment that were significantly associated with moderate-to-severe diarrhoea (MSD) at the GEMS Kenya site [5].

d Adjusted for age group and sex, stratified estimates by age group and/or sex presented where effect modification significant at 0.05 was observed.

e Water source that does not meet the criteria for ‘improved’, per the Joint Monitoring Program criteria [40] of a source that is safely protected from outside contamination (especially faeces) via its construction or intervention.

f Effective water treatment classified as solar disinfection, chlorine disinfection, boiling or filtration through ceramic or other filter. Ineffective water treatment classified as filtration through a cloth, alum or other chemical added.

At 60-day follow-up, G1 + G3 controls had higher odds of having visited a health facility for diarrhoea (OR 4.8, 95% CI 3.7–6.3) or having had dysentery (OR 3.8, 95% CI 1.5–10.6) during the follow-up period than G4 controls. G1 + G3 controls also had higher odds of fever (OR 1.8, 95% CI 1.5–2.1) or of having visited a health facility for fever (OR 1.5, 95% CI 1.2–1.8) during the follow-up period than G4 controls. G2 and G4 controls did not differ significantly in health outcomes at follow-up.

Exclusion of the 125 (4.9%) control children with repeat enrolments did not appreciably change the results of our analyses (data not shown).

### Stunting in controls by presence of diarrhoea and detection of enteric pathogens, adjusted for age and sex

Controls that did and did not develop any diarrhoea did not vary significantly in odds of stunting at enrolment or follow-up (Table 5a). Controls with MSD enteric pathogens detected in stool (both with and without diarrhoea) did not differ from controls without an MSD enteric pathogen in odds of being stunted at enrolment, but had significantly higher odds of being stunted at 60-day follow-up (OR 1.6, 95% CI 1.1–2.2, Table 5b). Conversely, controls with any potential pathogen detected in stool had significantly higher odds of being stunted at enrolment (OR 1.3, 95% CI 1.1–1.6), but not at 60-day follow-up, compared with controls without a potential pathogen detected. Controls did not differ in odds of stunting by G1–4 designations of diarrhoea/MSD pathogen status (Table 5c).

**Table 5.** Odds ratios for stunting among controls by diarrhoea and enteric pathogen detection in stool, Global Enteric Multicenter Study, Kenya site^a^

(a) Reported diarrhoea			
*Diarrhoea* + /−	Any diarrhoea reported *n* = 919		No diarrhoea reported *n* = 1465
Enrolment	1.10 (0.92, 1.33)		Ref.
60-day follow-up	0.99 (0.73, 1.34)		Ref.
(b) Pathogen detection in stool			
*MSD pathogen*^b^ + /−	⩾1 MSD pathogens detected *n* = 460		0 MSD pathogens detected *n* = 1924
Enrolment	1.12 (0.89, 1.41)		Ref.
60-day follow-up	**1.57 (1.09, 2.23)**		Ref.
*Any potential enteric pathogen*^c^ + /−	1 + potential pathogens detected *n* = 1629		0 enteric pathogens detected *n* = 755
Enrolment	**1.29 (1.06, 1.57)**		Ref.
60-day follow-up	1.14 (0.84, 1.57)		Ref.
(c) Diarrhoea/MSD enteric pathogen groups (G1–4)			
*Diarrhoea and MSD enteric pathogen* + /−	G1 + G3^d^ Diarrhoea *n* = 919	G2 No diarrhoea, 1 + MSD pathogen^b^ detected *n* = 262	G4 No diarrhoea, 0 MSD pathogens^b^ detected *n* = 1203
Enrolment	1.11 (0.91, 1.34)	1.01 (0.74, 1.36)	Ref.
60-day follow-up	1.04 (0.76, 1.43)	1.31 (0.81, 2.08)	Ref.

**Bold** indicates significance at 0.05 level.

a Multivariable logistic regression models used for stunting outcomes. All models are adjusted for age group and sex. Models at 60-day follow-up include stunting at baseline as a predictor as well.

b Any pathogens detected in a child's stool specimen at enrolment that were significantly associated with moderate-to-severe diarrhoea (MSD) at the GEMS Kenya site [5].

c Any pathogens detected from the entire list of potential pathogens assessed in GEMS [22].

d G1 and G3 were combined to represent all control children for whom diarrhoea was reported in the 14 days following enrolment.

## Discussion

Among control children in the GEMS Kenya site, we found significant carriage of enteric pathogens associated with MSD (19%) and of any potential enteric pathogen (68%) at enrolment, and high incidence of diarrhoea soon after enrolment (27% within 7 days, 39% within 14 days). At follow-up, 28% of controls that reported developing diarrhoea on the memory aid had sought healthcare for diarrhoea, compared with only 7% of controls who had not reported developing diarrhoea. No data were collected that would allow episodes of diarrhoea among controls to be classified as MSD, but some that were severe enough to warrant a visit to a health facility may have met the GEMS case criteria. Controls with enteric pathogens detected in stool – with or without diarrhoea – had higher odds of stunting than those that did not have an enteric pathogen detected, suggesting analysis of such longer term outcomes may require case definitions inclusive of mild diarrhoea or subclinical infections.

This study is the first, to our knowledge, to separately examine the gastrointestinal health – including symptomatic and subclinical infection – of study controls at enrolment, during the 14 days following enrolment, and at 60-day follow-up. Eligibility criteria for GEMS controls required the child to have been free from diarrhoea in the preceding 7 days, as is common practice in case–control studies of diarrhoea [7–9, 11, 12]. Few case–control studies have collected such detailed data on a reference population, including (a) stool specimens at enrolment tested for the same comprehensive panel of enteric pathogens as case stool specimens; (b) a daily record of diarrhoea during the 14 days post-enrolment; and (c) 60-day follow-up visits to repeat anthropometric measurements and enquire about illness subsequent to enrolment. These additional data allow a more detailed characterisation of the referent population than is usually afforded.

The prevalence of enteric pathogens detected at enrolment and incidence of diarrhoea following enrolment suggest that a substantial proportion of this control population had either residual or incubating subclinical infection during the study period [27]. Alternatively, certain enteric pathogens detected in control stool specimens (e.g. ETEC or EPEC) may have ‘colonised’ the large intestine but lacked the signals within the intestinal environment required to activate virulence gene expression or previously acquired infection-derived immunity [6]. The high incidence of diarrhoea shortly after enrolment is an important indicator of active infection that may have been incubating at enrolment: in particular, in the 27% of controls who had diarrhoea within 7 days after enrolment (70% of controls with diarrhoea) and especially in the 10% of all controls who visited a health facility for diarrhoea between enrolment and follow-up. Controls who developed developing diarrhoea and experienced subsequent symptoms (fever, dysentery) that led them to seek care at a health centre could have had other host or environmental factors that predisposed them to more symptomatic or recurrent diarrhoea, besides the diarrhoeal episode reported on the memory. However, without repeat faecal microbiology at the time of diarrhoea onset and a comparator population that allows for adjustment of potential confounders, a causal relationship between reported diarrhoea on the memory aid and subsequent symptoms (fever) and care-seeking at the 60-day follow-up visit cannot be determined with certainty.

Data on the frequency of detection of each enteric pathogen, and on episodes of diarrhoea in controls, are necessary to more precisely identify risk factors for diarrhoeal pathogen-specific illness, and to estimate the fraction of MSD attributable to each pathogen. GEMS investigators applied enteric pathogen prevalence data from controls in pathogen-specific attribution estimates [5, 28], but data on the frequency of diarrhoea among controls have not yet been used to improve their accuracy. Controls found to have evidence of recent infection are often excluded from risk factor analyses [29, 30] to avoid misclassification and bias towards the null. Although total MSD pathogen carriage among controls was 19%, carriage of any single pathogen associated with MSD in the GEMS Kenya site did not exceed 5%, suggesting little risk of bias in the original calculations of attributable fraction.

Recent evidence suggests that subclinical enteric infections may have detrimental effects on long-term development in children, such as stunting, independent of diarrhoea [3, 31]. Data from this study are consistent with this previous evidence: controls with carriage of any potential enteric pathogen had a higher odds of stunting at enrolment compared with those without carriage of any potential enteric pathogen; those with carriage of MSD pathogens had a higher odds of stunting at follow-up compared with those without carriage of MSD pathogens, while reported diarrhoea was not significantly associated with stunting among controls. While interpretation of differences from our study is limited given the case–control study design and short follow-up period (60 days), previous evidence suggests that repeat symptomatic and subclinical infections may lead to environmental enteric dysfunction (EED), a state of chronic inflammation of the gut [3, 32–35]. Evidence that EED may act independently of diarrhoea prevalence has been observed in studies employing a longer follow-up period [36], including a multisite birth cohort of children 0–2 years of age [37]. These data, combined with results from this study, suggest assessment of enteric pathogen carriage should accompany measurement of diarrhoea when evaluating long-term outcomes such as linear growth.

Timing of outcome onset may be important in reducing outcome misclassification, as up to 10% of all controls (including >25% of controls reporting diarrhoea) in this analysis may have qualified as cases within the 60-day follow-up period. Because extending the period when potential controls must be absent diarrhoea prior to enrolment may be both logistically challenging and present concerns of recall bias, an alternative strategy of disaggregating controls into subgroups based on clinical variation may be more feasible, with subsequent analysis targeting symptom- and pathogen-free controls as necessary.

This study has limitations. First, because GEMS is a tightly matched case–control study, the results from controls are not generalisable to the entire study population, and implications should be limited to reference populations in case–control studies. Second, detection of enteric pathogens in stool at enrolment does not provide information about the timing or association with symptom onset, limiting conclusions about the aetiologic cause of reported diarrhoea. Though reported diarrhoea has a well-documented potential for bias with varying recall periods [14–16], use of a memory-aid form filled daily [18] may have minimised these issues. However, reduced incidence of reported diarrhoea in the second week of memory aid documentation (Fig. 1) may also suggest that caregivers’ adherence to filling the form decreased over time. Of note, the use of laboratory tests with high sensitivity to potentially low pathogen loads in individuals without diarrhoea (e.g. controls) in GEMS was a study strength [27].

It is important that future studies of enteric infection and diarrhoea, especially case–control designs like GEMS, continue to employ sensitive enrolment and follow-up measures – including potential assessment of underlying or subsequent subclinical enteric infections through molecular diagnostics – to minimise misclassification and contextualise study results with regard to background levels of infection. Given recent progress in diagnostic techniques, including multiplex polymerase chain reaction [38, 39], improved characterisation of study outcomes from stool specimens is becoming more feasible in low- and middle-income countries. Additionally, the use of a memory-aid form or other, similar method may improve capture of symptom onset after enrolment [18].

This analysis of control children in the GEMS Kenya site, who reported no diarrhoea in the week preceding enrolment, revealed that many had underlying residual, concurrent or incubating enteric infection or colonisation. Some of these may have been subclinical infections and a significant number went on to have diarrhoea in the following 2 weeks. Odds of stunting varied significantly by detection of enteric pathogens in stool, regardless of diarrhoeal symptoms, which is in agreement with other, multisite birth cohort studies [37] underscoring the importance of measuring enteric pathogen carriage in stool in addition to diarrhoeal outcomes. This variation in both short- and long-term health outcomes in control children underscores the importance of extending the use of sensitive metrics for case status to controls to better understand their health status and more accurately characterise the study reference group.

## Supplementary material

For supplementary material accompanying this paper visit https://doi.org/10.1017/S0950268818002972. click here to view supplementary material
