# Supplementary material for: Diarrhoea, enteric pathogen detection and nutritional indicators among controls in the Global Enteric Multicenter Study, Kenya site: an opportunity to understand reference populations in case–control studies of diarrhoea
Source: Epidemiol Infect. 2018 Nov 15;147:e44. doi: 10.1017/S0950268818002972 (PMC6518569; doi:10.1017/S0950268818002972)
Supplement: Supplementary file 1 [file S0950268818002972sup001.docx]

| **Table S1**  a. Distribution of participants who did/did not fill out memory aid and had/did not have stool collected for enteric infection detection | | | |
| --- | --- | --- | --- |
|  | Stool collected | Stool not collected | Total |
| Filled out memory aid | 2384 | 0 | 2384 |
| Did not fill out memory aid | 150 | 0 | 150 |
| Total | 2534 | 0 | 2534 |
|  |  |  |  |
| b. Distribution of participants with and without MSD enteric pathogen^1^ detected/diarrhea | | | |
|  | Diarrhea reported | No diarrhea reported | Total |
| MSD enteric pathogen detected | 198 | 262 | 460 |
| No enteric pathogen detected | 721 | 1203 | 1,924 |
| Total | 919 | 1465 | 2384 |

^1^Any pathogens detected in a child’s stool specimen at enrollment that were significantly associated with moderate-to-severe diarrhea (MSD) at the GEMS Kenya site [5].

| Table S2: Prevalence of enteric pathogens in stool collected from controls with/without any diarrhea in 14-day memory aid form, Global Enteric Multicenter Study, Kenya site^1^ | | | | |
| --- | --- | --- | --- | --- |
| Pathogen detection | Controls with any diarrhea  n=919 | Controls without any diarrhea  n=1465 | OR (95% CI) | p-value |
| No pathogen detected (MSD^2^) | 721 (78.5%) | 1203 (82.1%) |  |  |
| No pathogen detected (any^3^) | 284 (30.9%) | 471 (32.2%) |  |  |
| Only 1 pathogen detected (MSD^2^) | 182 (19.8%) | 245 (16.7%) |  |  |
| Only 1 pathogen detected (any^3^) | 380 (41.3%) | 605 (41.3%) |  |  |
| 2+ pathogens detected (MSD^2^) | 16 (1.7%) | 17 (1.2%) |  |  |
| 2+ pathogens detected (any^3^) | 255 (27.8%) | 389 (26.5%) |  |  |
| Bacteria |  |  |  |  |
| *ST-ETEC | 42 (4.6%) | 58 (4.0%) | 1.12 (0.74, 1.69) | 0.576 |
| LT-ETEC | 57 (6.2%) | 80 (5.5%) |  |  |
| Females |  |  | 0.71 (0.40, 1.25) | 0.237 |
| Males |  |  | 1.48 (0.93, 2.34) | 0.099 |
| EAEC | 161 (17.5%) | 229 (15.6%) | 1.08 (0.86, 1.35) | 0.521 |
| *tEPEC | 56 (6.1%) | 59 (4.0%) | 1.45 (0.99, 2.12) | 0.054 |
| aEPEC | 56 (6.1%) | 94 (6.4%) | 0.96 (0.68, 1.35) | 0.810 |
| EHEC | 0 | 0 | - | - |
| **Shigella* spp. | 20 (2.2%) | 32 (2.2%) |  |  |
| Females |  |  | 0.41 (0.14, 1.23) | 0.113 |
| Males |  |  | 1.73 (0.83, 3.59) | 0.140 |
| *Aeromonas* | 1 (0.1%) | 3 (0.2%) | 0.52 (0.03, 4.14) | 0.576 |
| *V. cholerae O1* | 0 | 0 | - | - |
| *S.* Typhi | 0 | 0 | - | - |
| *Non-typhoidal *Salmonella* | 37 (4.0%) | 43 (2.9%) | 1.31 (0.83, 2.06) | 0.236 |
| *C. jejuni* | 82 (8.9%) | 105 (7.2%) | 1.24 (0.91, 1.67) | 0.171 |
| *C. coli* | 45 (4.9%) | 91 (6.2%) | 0.74 (0.51, 1.07) | 0.111 |
|  |  |  |  |  |
| Viruses |  |  |  |  |
| *Rotavirus | 23 (2.5%) | 26 (1.8%) | 1.33 (0.75, 2.36) | 0.322 |
| GI Norovirus | 36 (3.9%) | 59 (4.0%) | 1.00 (0.65, 1.52) | 0.999 |
| GII Norovirus | 36 (3.9%) | 60 (4.1%) | 0.89 (0.58, 1.35) | 0.591 |
| Adenovirus 40/41 | 12 (1.3%) | 8 (0.5%) | 2.12 (0.87, 5.44) | 0.102 |
| Adenovirus non-40/41 | 21 (2.3%) | 36 (2.5%) | 0.88 (0.50, 1.51) | 0.649 |
| Astrovirus | 11 (1.2%) | 26 (1.8%) | 0.72 (0.34, 1.44) | 0.372 |
| Sapovirus | 26 (2.8%) | 45 (3.1%) | 0.90 (0.54, 1.47) | 0.688 |
|  |  |  |  |  |
| Protozoa |  |  |  |  |
| *Giardia* | 200 (21.8%) | 364 (24.8%) | 0.90 (0.73, 1.10) | 0.311 |
| **Cryptosporidium* | 36 (3.9%) | 62 (4.2%) | 0.86 (0.56, 1.30) | 0.477 |
| *E. histolytica* | 2 (0.2%) | 5 (0.3%) | 0.59 (0.08, 2.76) | 0.530 |

^1^All odds ratios (ORs) and 95% confidence intervals (95% CI) adjusted for age group and sex, with age- or sex-specific stratified estimates presented where effect modification was significant at 0.05. ^2^Any pathogens detected in a child’s stool specimen at enrollment that were significantly associated with moderate-to-severe diarrhea (MSD) at the GEMS Kenya site [5], also denoted by an asterisk (*) in table. ^3^Any pathogens detected from the entire list of potential pathogens assessed in GEMS [22].

| Table S3: Prevalence of enteric pathogens in stool collected from controls with/without any diarrhea within 7 days of enrollment in 14-day memory aid form, Global Enteric Multicenter Study, Kenya site^1^ | | | | |
| --- | --- | --- | --- | --- |
| Pathogen detection | Controls with any diarrhea within 7d of enrollment  n=643 | Controls without any diarrhea within 7d of enrollment  n=1741 | OR (95% CI) | p-value |
| No MSD pathogen^2^ detected | 501 (77.9%) | 1,408 (81.7%) |  |  |
| Only 1 MSD pathogen^2^ detected | 131 (20.4%) | 294 (17.1%) |  |  |
| 2+ MSD pathogens^2^ detected | 11 (1.7%) | 22 (1.3%) |  |  |
| Bacteria |  |  |  |  |
| *ST-ETEC | 34 (5.3%) | 66 (3.8%) | 1.40 (0.90, 2.12) | 0.123 |
| LT-ETEC | 41 (6.4%) | 96 (5.5%) |  |  |
| Females |  |  | 0.60 (0.30, 1.17) | 0.131 |
| Males |  |  | **1.67 (1.04, 2.69)** | **0.034** |
| EAEC | 114 (17.7%) | 276 (15.9%) | 1.07 (0.84, 1.36) | 0.582 |
| *tEPEC | 36 (5.6%) | 79 (4.5%) | 1.19 (0.78, 1.77) | 0.406 |
| aEPEC | 38 (5.9%) | 112 (6.4%) | 0.93 (0.63, 1.35) | 0.703 |
| EHEC | 0 (0%) | 0 (0%) | - | - |
| **Shigella* spp. | 14 (2.2%) | 38 (2.2%) | 1.05 (0.54, 1.90) | 0.886 |
| *Aeromonas* | 1 (0.2%) | 3 (0.2%) | 0.92 (<0.01, 7.24) | 0.943 |
| *V. cholerae O1* | 0 (0%) | 0 (0%) | - | - |
| *S.* Typhi | 0 (0%) | 0 (0%) | - | - |
| *Non-typhoidal *Salmonella* | 28 (4.4%) | 52 (3.0%) |  |  |
| 0-11 mo |  |  | 0.91 (0.46, 1.81) | 0.797 |
| 12-23 mo |  |  | 1.49 (0.62, 3.56) | 0.373 |
| 24-59 mo |  |  | **3.87 (1.38, 10.8)** | **0.010** |
| *C. jejuni* | 56 (8.7%) | 131 (7.5%) | 1.14 (0.81, 1.57) | 0.449 |
| *C. coli* | 31 (4.8%) | 105 (6.0%) | 0.78 (0.51, 1.16) | 0.237 |
|  |  |  |  |  |
| Viruses |  |  |  |  |
| *Rotavirus | 18 (2.8%) | 31 (1.8%) | 1.55 (0.84, 2.77) | 0.146 |
| GI Norovirus | 27 (4.2%) | 68 (3.9%) | 1.09 (0.68, 1.71) | 0.702 |
| GII Norovirus | 26 (4.0%) | 70 (4.0%) | 0.96 (0.59, 1.50) | 0.849 |
| Adenovirus 40/41 | 9 (1.4%) | 11 (0.6%) | 2.06 (0.82, 5.01) | 0.111 |
| Adenovirus non-40/41 | 14 (2.2%) | 43 (2.5%) | 0.88 (0.46, 1.58) | 0.678 |
| Astrovirus | 11 (1.7%) | 26 (1.5%) | 1.24 (0.58, 2.47) | 0.557 |
| Sapovirus | 16 (2.5%) | 55 (3.2%) | 0.79 (0.44, 1.37) | 0.424 |
|  |  |  |  |  |
| Protozoa |  |  |  |  |
| *Giardia* | 135 (21.0%) | 429 (24.6%) |  |  |
| 0-11 mo |  |  | 1.17 (0.74, 1.84) | 0.497 |
| 12-23 mo |  |  | **0.63 (0.43, 0.92)** | **0.016** |
| 24-59 mo |  |  | 0.99 (0.69, 1.42) | 0.947 |
| **Cryptosporidium* | 23 (3.6%) | 75 (4.3%) | 0.78 (0.48, 1.24) | 0.316 |
| *E. histolytica* | 1 (0.2%) | 6 (0.3%) | 0.42 (0.02, 2.48) | 0.423 |

^1^All odds ratios (ORs) and 95% confidence intervals (95% CI) adjusted for age group and sex, with age- or sex-specific stratified estimates presented where effect modification was significant at 0.05. ^2^Any pathogens detected in a child’s stool specimen at enrollment that were significantly associated with moderate-to-severe diarrhea (MSD) at the GEMS Kenya site [5], also denoted by an asterisk (*) in table.

| Table S4: Analysis of controls with/without potential enteric pathogens detected in stool at enrollment, Global Enteric Multicenter Study, Kenya site | | | | |
| --- | --- | --- | --- | --- |
| Parameter | 1+ potential enteric pathogens detected^1^  N = 1,629 | 0 potential enteric pathogens detected^1^  N = 755 | aOR^2^ | p-value^2^ |
| a) Health conditions at enrollment |  |  |  |  |
| Blood in stool collected | 3 (0.2%) | 1 (0.1%) | 1.43 (-) | 0.758 |
| Blood in stool (in last 7 days) | 6 (0.3%) | 1 (0.1%) | 2.29 (0.37, 44.0) | 0.451 |
| Fever (in last 7days) | 648 (37.3%) | 295 (37.1%) | 1.00 (0.83, 1.19) | 0.983 |
| Vomiting (in last 7days) | 49 (2.8%) | 22 (2.8%) | 1.00 (0.60, 1.70) | 0.985 |
|  |  |  |  |  |
| b) Water, sanitation, and hygiene conditions at enrollment |  |  |  |  |
| Any sanitation facility present | 1329 (76.4%) | 589 (74.1%) | 1.17 (0.96, 1.43) | 0.115 |
| Unimproved water source^3^ | 635 (36.5%) | 278 (35.0%) | 1.09 (0.91, 1.31) | 0.335 |
| Water treated | 972 (55.9%) | 452 (56.9%) | 0.98 (0.83, 1.17) | 0.849 |
| Water treated effectively^4^ | 916 (52.7%) | 429 (54.0%) | 0.96 (0.81, 1.15) | 0.685 |
| Water treated with chlorine | 782 (45.0%) | 362 (45.5%) | 0.99 (0.84, 1.18) | 0.952 |
|  |  |  |  |  |
| c) Health at 60d follow-up |  |  |  |  |
| Diarrhea | 698 (41.2%) | 333 (43.0%) | 0.89 (0.74, 1.06) | 0.189 |
| Visited health facility for diarrhea in last 60 day | 256 (14.7%) | 122 (15.3%) | 0.92 (0.72, 1.18) | 0.504 |
| Dysentery in last 60d | 18 (1.1%) | 7 (0.9%) | 1.15 (0.49, 3.00) | 0.751 |
| Visited health facility for dysentery in last 60 day | 9 (0.5%) | 4 (0.5%) | 0.93 (0.29, 3.50) | 0.904 |
| Fever in last 60 day | 974 (57.5%) | 445 (57.4%) |  |  |
| 0-11 mo |  |  | 1.26 (0.94, 1.70) | 0.123 |
| 12-23 mo |  |  | 0.95 (0.69, 1.29) | 0.730 |
| 24-59 mo |  |  | 0.80 (0.59, 1.09) | 0.157 |
| Visited health facility for fever in last 60day | 319 (18.3%) | 149 (18.7%) | 0.99 (0.79, 1.24) | 0.935 |
| Death of child | 12 (0.7%) | 3 (0.4%) | 2.37 (0.63, 15.4) | 0.262 |

**Bold** indicates significant at 0.05. Multivariable logistic regression used for all parameters. ^1^Based on stool specimen collected at enrollment, defined as any pathogens detected from the entire list of potential pathogens assessed in GEMS [22] ^2^Adjusted for age group and sex, stratified estimates by age group or sex presented where effect modification significant at 0.05 was observed; ^3^Water source that does not meet the criteria for “improved,” per the Joint Monitoring Program criteria [40] of a source that is safely protected from outside contamination (especially feces) via its construction or intervention ^4^Effective water treatment classified as solar disinfection, chlorine disinfection, boiling, or filtration through ceramic or other filter. Ineffective water treatment classified as filtration through a cloth, alum, or other chemical added;

| Table S5: Health and WASH conditions among controls by enteric pathogen detection in stool and diarrhea, Global Enteric Multicenter Study, Kenya site | | | | | |
| --- | --- | --- | --- | --- | --- |
|  | Group 1 (G1) | Group 2 (G2) | Group 3 (G3) | Group 4 (G4) |  |
| Parameter | Diarrhea, ≥1 MSD pathogen^1^ detected  n=198 | No diarrhea, ≥1 MSD enteric pathogen^1^ detected  n=262 | Diarrhea, 0 MSD pathogens^1^ detected  n = 721 | No diarrhea, 0 MSD pathogens^1^ detected  n=1203 |  |
| Health conditions at enrollment |  |  |  |  |  |
| Median number of pathogens detected (range) | 1 (1-2) | 1 (1-3) | 0 | 0 |  |
| Blood in stool collected | 0 (0%) | 0 (0%) | 0 (0%) | 4 (0.3%) |  |
| Blood in stool (in last 7 days) | 0 | 1 (0.4%) | 2 (0.3%) | 3 (0.2%) |  |
| Fever (in last 7 days) | 85 (42.9%) | 94 (35.9%) | 316 (43.8%) | 386 (32.1%) |  |
| Vomiting (in last 7 days) | 5 (2.5%) | 11 (4.2%) | 28 (3.9%) | 25 (2.1%) |  |
|  |  |  |  |  |  |
| WASH conditions at enrollment |  |  |  |  |  |
| Any sanitation facility present | 150 (75.8%) | 209 (79.8%) | 547 (75.9%) | 897 (74.6%) |  |
| Unimproved water source^2^ | 79 (40.3%) | 101 (39.0%) | 277 (38.5%) | 444 (37.2%) |  |
| Water treated | 109 (55.6%) | 157 (60.6%) | 411 (57.1%) | 662 (55.5%) |  |
| Water treated effectively^3^ | 109 (55.1%) | 149 (56.9%) | 387 (53.7%) | 622 (51.7%) |  |
| Water treated with chlorine | 90 (45.5%) | 131 (50.0%) | 343 (47.6%) | 517 (43.0%) |  |
|  |  |  |  |  |  |
| Health conditions at 60-day follow-up |  |  |  |  |  |
| Visited health facility for diarrhea | 60 (30.3%) | 17 (6.5%) | 193 (26.8%) | 84 (7.0%) |  |
| Dysentery | 3 (1.5%) | 2 (0.8%) | 13 (1.8%) | 6 (0.5%) |  |
| Visited health facility for dysentery | 1 (0.5%) | 0 (0%) | 7 (1.0%) | 4 (0.3%) |  |
| Fever | 129 (65.8%) | 125 (48.3%) | 477 (66.3%) | 620 (52.0%) |  |
| Visited health facility for fever | 42 (21.2%) | 37 (14.1%) | 167 (23.2%) | 197 (16.4%) |  |
| Death of child | 5 (2.6%) | 1 (0.4%) | 3 (0.4%) | 4 (0.3%) |  |

^1^Any pathogens detected in a child’s stool specimen at enrollment that were significantly associated with moderate-to-severe diarrhea (MSD) at the GEMS Kenya site [5]. ^2^Water source that does not meet the criteria for “improved,” per the Joint Monitoring Program criteria [40] of a source that is safely protected from outside contamination (especially feces) via its construction or intervention ^3^Effective water treatment classified as solar disinfection, chlorine disinfection, boiling, or filtration through ceramic or other filter. Ineffective water treatment classified as filtration through a cloth, alum, or other chemical added
